# Commentary: The research progress of Chikungunya fever

**DOI:** 10.3389/fpubh.2025.1681894

**Published:** 2025-11-21

**Authors:** Jiayi Chen

**Affiliations:** Department of Stomatology, Suzhou Wujiang District Hospital of Traditional Chinese Medicine, Suzhou, China

**Keywords:** Chikungunya, pathogenesis, vaccine, epidemiology, prevention

The mini review by Cai et al. (1) in *Frontiers in Public Health* provided a timely and comprehensive synthesis of the current understanding of Chikungunya virus (CHIKV), covering epidemiology, pathogenesis, and vaccine development. This is particularly relevant as CHIKV continues to expand beyond historically endemic regions, driven by vector adaptation, globalization, and climate change (2).

Chikungunya fever is an acute infectious disease caused by the Chikungunya virus (CHIKV) and transmitted by Aedes mosquitoes. CHIKV is a positive sense, single-stranded RNA (ssRNA) virus with an 11.8 kbp size of the genome, which consists of 1,244 amino acids structural and 2,472 amino acids nonstructural polyprotein (3). It is mainly characterized by fever, rash and joint pain. Globally, there are 35 million annual infections, mainly in Southeast Asia, Africa and the Americas. A global disease burden of Chikungunya virus infections revealed that 2.8 billion people experienced Chikungunya fever across 104 countries, and the mean duration between outbreaks was 6.2 years, with 8.4% of the susceptible population infected per outbreak (4). Usually, CHIKV viral load is associated with disease prognosis and the limit of detection is500 copies/μl using qRT-PCR. A virological analysis from India revealed that patients with higher initial viral loads during the acute phase of illness had poor prognosis at the post-acute phase with more restricted joint movement and higher visual analog score (VAS) (5).

A key strength of Cai et al.'s work lay in linking molecular mechanisms—such as MXRA8-mediated viral entry and apoptosis-driven immune evasion—with the spectrum of clinical outcomes, ranging from acute febrile illness to chronic arthralgia. This integrative approach was critical for identifying novel therapeutic targets and informing clinical management. Their detailed account of vaccine platforms, including live-attenuated, inactivated, recombinant vector, DNA, and mRNA candidates, underscored the breadth of global research efforts.

Nonetheless, several aspects merit further emphasis. First, the challenge of accurate diagnosis remains substantial, particularly in resource-limited settings where CHIKV is often clinically indistinguishable from dengue or Zika virus infection (6). The expansion of rapid molecular diagnostics and multiplex assays would improve outbreak detection and surveillance accuracy. Second, integrated vector management should be advanced alongside biomedical solutions. Interventions such as *Wolbachia*-based biological control and targeted environmental management have shown promise in reducing Aedes mosquito populations and, consequently, arboviral transmission (7).

Cai et al. also noted the looming threat posed by climate change. Incorporating predictive models that integrated climate, land use, and vector ecology could identify emerging hotspots and inform pre-emptive interventions (8). Such models, coupled with community-based vector control and vaccine rollout strategies, could form the backbone of a proactive CHIKV preparedness framework.

In conclusion, the review by Cai et al. represented an important contribution to the field. Strengthening diagnostic capacity, implementing multi-pathogen vector control, and integrating climate-informed risk assessments into public health policy will be crucial for reducing the global burden of CHIKV in the coming decades.
